# Autistic traits predict poor integration between top-down contextual expectations and movement kinematics during action observation

**DOI:** 10.1038/s41598-018-33827-8

**Published:** 2018-11-01

**Authors:** L. Amoruso, A. Finisguerra, C. Urgesi

**Affiliations:** 1 Laboratory of Cognitive Neuroscience, Department of Languages and Literatures, Communication, Education and Society, University of Udine, Udine, Italy; 2 Basque Center of Cognition, Brain and Language, San Sebastian, Spain; 3 Scientific Institute, IRCCS Eugenio Medea, Pasian di Prato, Udine, Italy

## Abstract

Autism is associated with difficulties in predicting and understanding other people’s actions. There is evidence that autistic traits are distributed across a spectrum and that subclinical forms of autistic impairments can also be measured in the typical population. To investigate the association between autistic traits and motor responses to others’ actions, we quantified these traits and measured cortico-spinal excitability modulations in M1 during the observation of actions embedded in congruent, incongruent and ambiguous contexts. In keeping with previous studies, we found that actions observed in congruent contexts elicited an early facilitation of M1 responses, and actions observed in incongruent contexts, resulted in a later inhibition. Correlational analysis revealed no association between autistic traits and the facilitation for congruent contexts. However, we found a significant correlation between motor inhibition and autistic traits, specifically related to social skills and attention to details. Importantly, the influence of these factors was independent from each other, and from the observer’s gender. Thus, results suggest that individuals with higher social deficits and greater detail-processing style are more impaired in suppressing action simulation in M1 when a mismatch between kinematics and context occurs. This points to difficult integration between kinematics and contextual representations in the autistic-like brain.

## Introduction

Comprehending the intentions underlying other people’s actions from observing their movements constitutes a crucial ability in humans’ everyday-life. This ability is thought to rely on the mirror neuron system (MNS), a fronto-parietal network that is active during both the observation and the execution of the same or similar action^1^. Furthermore, there is evidence suggesting that this ability may be anticipatory in nature, enabling for predictive coding of other’s behaviors^2–5^.

Socio-motor impairments, including difficulties in action prediction and comprehension, are considered to be among the core deficits associated with Autism Spectrum Disorders (ASD)^6^. It has been argued^7^ that these impairments could be explained in terms of a global dysfunction in the MNS (i.e., the broken mirror theory of ASD).

Evidence of mirror-like activity in humans comes from single-pulse transcranial magnetic stimulation (spTMS) studies measuring motor facilitation over the primary motor cortex (M1) during action observation. In these studies, the amplitude of TMS-induced motor-evoked potentials (MEPs) are taken as an index of mirror responses in fronto-parietal areas, which enhance the excitability of M1 via cortico-cortical connections^8,9^. To date, only a few studies have tested the broken mirror theory hypothesis using spTMS, yielding contradictory results. For instance, Theoret, Halligan^10^ found reduced motor facilitation in individuals with ASD as compared to neurotypical ones while observing meaningless hand movements. Enticott, Kennedy^11^ showed that, during the observation of transitive hand gestures, ASD individuals presented a significant reduction in M1 responses as compared to controls. However, in a subsequent study in which they used different experimental stimuli (individual vs. interactive hand actions), comparable motor responses were observed in both, ASD and neurotypical individuals^12^. Furthermore, neuroimaging studies also provide contradictory evidence on whether the MNS is compromised in ASD, with studies either reporting preserved^13,14^ or abnormal activity^15,16^ within this network. Thus, overall, a dysfunction in the MNS, by itself, seems to be insufficient in providing a complete account of ASD symptomatology.

To get an insight into the possible reasons for this discrepancy, it is important to note that socio-motor deficits in ASD have been traditionally studied by using non-realistic stimuli (i.e., meaningless movements, snapshots of hands detached from background). These stimuli are not good replicas of the world because they lack the context of everyday-life situations^17^. Indeed, body movements are not perceived in isolation but with objects, actors, and the relationships among them ‘gluing together’ into a unifying scene. In other words, this integrative aspect, which is inherent to the processing of naturalistic scenes, seems to be missing in most previous studies measuring action comprehension in ASD.

When considered in light of recent models that emphasize the integrative nature of ASD deficits, this aspect become even more critical. For instance, it has been recently suggested^18^ that the MNS might be modulated by top-down social signals from prefrontal regions and that a failure in this integration could explain ASD socio-motor impairments. Similarly, it has been proposed that ASD individuals could suffer from a deficit in integrating low sensory perceptual aspects and high level intentional representations during action comprehension^19^. Thus, according to these models, ASD impairments would arise when integrating perceptual evidence with top-down signals. In a similar vein, recent appealing proposals based on the Bayesian framework suggest that autistic observers would fail in optimally combining current sensory evidence with prior expectations and contextual information regarding the likely causes of input^20,21^.

Interestingly, there is evidence that individuals with subclinical ASD^22^ exhibit a similar profile (i.e., stronger reliance on the sensory evidence and less sensitivity to context), thus suggesting that autistic-like impairments are also measurable, albeit at lower levels of severity, in neurotypical individuals^23,24^.

Here, we examined whether individual differences in autistic-like traits relate to the ability of combining perceptual information about observed actions (i.e., perceptual kinematics) with prior expectations derived from naturalistic contexts. We capitalized on previous studies^25–27^ showing that, during an action prediction task, the observation of kinematics occurring in congruent contexts facilitated M1 excitability at early stages (~300 ms after action onset), while the observation of kinematics in incongruent contexts resulted in a later inhibition of motor facilitation (~500 ms after action onset). These results were interpreted as reflecting the interplay between simulative motor resonance responses triggered by the observation of low-level movement kinematics and top-down signals generated in areas beyond the MNS and conveying information about the intention predicted from the context.

**Figure 1:**
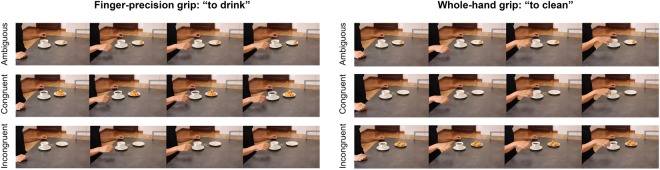
Examples of stimuli and conditions. Depending on the action, the reach-to-grasp movement kinematics was different in terms of precision vs. whole-hand grips. In addition, the actions could be performed in three different contexts: congruent, incongruent, and ambiguous.

In the current study, we used the same task and similar procedures as in previous studies^25–27^. More specifically, we recorded MEPs from the FDI and a control muscle in 22 participants while they observed videos of everyday actions embedded in congruent, incongruent or ambiguous contexts. Videos were interrupted before action ending and participants were asked to predict how action will unfold. TMS pulse could be delivered at either 300 ms or 500 ms after action onset. Congruency between action and context was manipulated in terms of compatibility between the pattern of observed movement kinematics and the scenario in which it was embedded. For instance, one of the videos depicted a breakfast scenario (i.e., a mug full of coffee and a plate with some biscuits). If the model grasped the mug by its handle with a precision grip, this condition was coded as congruent. However, if the mug was grasped using a whole-hand grip from the top, this condition was coded as incongruent, given that this type of grasping prevented the model from drinking in a context were the highly expected action was “to drink”. After showing the video, two possible descriptors (i.e., to drink and to clean) were presented on the screen and participants had to select, given contextual and kinematics information, which was the actor’s more likely intention. Importantly, we also included ambiguous contexts (i.e., a mug half full of coffee) in which both types of actions and associated grasping movements were equally plausible (see Fig. 1 and Videos 1–3 for examples of the stimuli used in the study).

We reasoned that this task suited well for testing whether the amount of autistic-like traits, as measured by the Autistic Quotient^28^, was associated with different modulation of motor responses according to the congruency between the observed kinematics and the context. Based on the “combinatorial models” suggesting that the integration between perceptual signals and prior or contextual knowledge is compromised in ASD, we hypothesized that during action recognition the individuals who exhibit higher levels of autistic-like traits would show impairments in effectively combining the sensory evidence conveyed by the perceptual kinematics with the overarching action goal representation estimated from the naturalistic context. More specifically, we expected that the high prevalence of autistic traits would be related to a reduction in the facilitatory and inhibitory effects for actions embedded, respectively, in congruent and incongruent contexts.

## Results

### Behavioral Results

**Figure 2:**
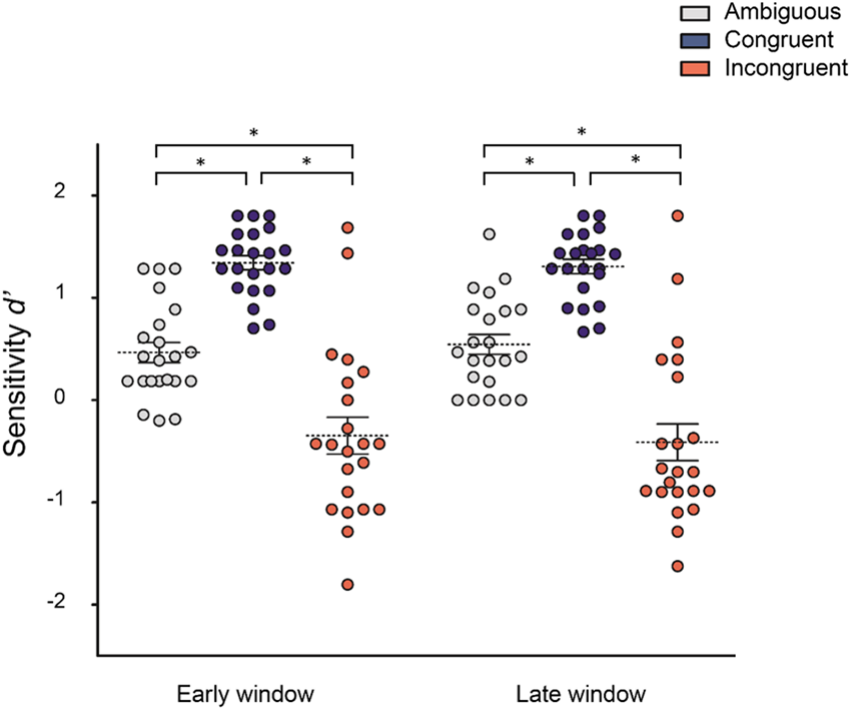
Behavioural results. Participants’ performance in predicting the course of the observed actions (expressed as *d*’) during the 3 action observation conditions (Congruent, Incongruent, and Ambiguous) at the 2 time-windows (early, late). Data points represent individual participants. Asterisks indicate significant comparison (*p* < 0.05). Error bars represent SEM and dotted lines MEAN.

Figure 2 shows participant’s *d* values for each context and time-point. The RM-ANOVA performed on these values yielded a significant main effect of context (*F*_2, 42_ = 78.88, *p* < 0.0001, ${\eta }_{p}^{2}$ = 0.78). Non-significant main effect of time (*p* = 0.88) or interactions between time and context were observed (*p* = 0.43). Post-hoc comparisons (MSE = 0.40, df = 42) showed that, across the two time-windows, kinematics embedded in congruent contexts (M = 1.32, SD = 0.32) was better recognized as compared to that observed in ambiguous (M = 0.50, SD = 0.45, *p* = 0.0001) or incongruent ones (M = −0.37, SD = 0.83, *p* = 0.0001). In addition, kinematics observed in ambiguous contexts was better recognized than that observed in incongruent ones (*p* = 0.0001).

### MEP Results

The raw mean amplitudes of MEPs recorded from the FDI and the ECR muscles in the different conditions are reported in Table 1.

**Table 1:** Mean raw MEP amplitudes for each experimental condition.

	Ambiguous		Congruent		Incongruent	
	FDI	ECR	FDI	ECR	FDI	ECR
300 ms	0.959 ± 0.148	0.511 ± 0.054	1.028 ± 0.159	0.468 ± 0.044	0.931 ± 0.141	0.496 ± 0.058
500 ms	0.839 ± 0.112	0.449 ± 0.049	0.962 ± 0.158	0.472 ± 0.048	0.740 ± 0.107	0.457 ± 0.048

Values corresponding to the raw amplitudes (mean ± SEM) of MEPs recorded from the FDI and the ECR muscles in the 3 observation conditions (Congruent, Incongruent, and Ambiguous) for the time-points of interest (300 ms and 500 ms).

The dependent-sample *t*-test (two-tailed) performed on the log-transformed amplitudes of MEPs recorded from the FDI and the ECR baseline blocks revealed no main effect of muscle (*t*_21_ = 1.68, *p* = 0.10), suggesting that MEPs recorded from both muscles were comparable. Thus, for each muscle, these baseline values were subtracted from the log-transformed MEPs during the action observation conditions to derive normalized index of motor facilitation.

To test how the intensity of motor facilitation of the two muscles was modulated by time and context, we entered normalized MEPs amplitudes into a 2 × 2 × 3 (muscle × time × context) RM-ANOVA. A significant intercept (*F*_1,21_ = 65.83, *p* < 0.0001, ${\eta }_{p}^{2}$ = 0.75) was found, ensuring that the overall motor facilitation index was different from 0. The omnibus RM-ANOVA revealed a main effect of muscle (*F*_1, 21_ = 12.86, *p* = 0.001, ${\eta }_{p}^{2}$ = 0.37), indicating that, overall, the FDI showed a greater activation as compared to the ECR muscle throughout the experiment. A main effect of context (*F*_2, 42_ = 3.79, p = 0.03, ${\eta }_{p}^{2}$ = 0.15) was also found, showing that the observation of actions in congruent contexts elicited a greater cortico-spinal excitability (CSE) facilitation as compared to those observed in incongruent ones. Finally, a main effect of time was observed (*F*_1, 21_ = 12.73, *p* = 0.001, ${\eta }_{p}^{2}$ = 0.37), indicating that CSE facilitation was greater in the early time window as compared to the later one. Furthermore, we found a significant muscle × time (*F*_1, 21_ = 8.05, *p* = 0.009, ${\eta }_{p}^{2}$ = 0.27), and muscle × context (*F*_2, 42 = _10.21, *p* = 0.0002, ${\eta }_{p}^{2}$ = 0.32) interactions, which were further qualified by a significant 3-way interaction of all variables (*F*_2, 42_ = 4.07, *p* = 0.024, ${\eta }_{p}^{2}$ = 0.16).

Post-hoc tests (MSE = 0.0005, df = 21) on the muscle × time interaction indicated that, despite the fact that CSE was greater for the FDI as compared to the ECR in both time windows (all *p*s < 0.001), both muscles showed increasing levels of CSE in the earlier time window and compared to the later one. Crucially, the post-hoc tests (MSE = 0.00053, df = 42) on the muscle × context, indicated that the FDI muscle (all *p*s < 0.004), but not the ECR muscle (all *p*s > 0.81), was modulated by context. Finally, post-hoc tests (MSE = 0.00024, df = 42) performed on the 3-way interaction showed that, in the earlier time-window (300 ms), motor facilitation for the FDI was enhanced for actions observed within a congruent context (M = 0.144, SD = 0.1) as compared to those observed in ambiguous (M = 0.131, SD = 0.09, *p* = 0.006) and incongruent ones (M = 0.126, SD = 0.09, *p* = 0.001), which in turn did not differ (*p* = 0.48). No modulations were observed for the ECR (all *p*s > 0.08).

**Figure 3:**
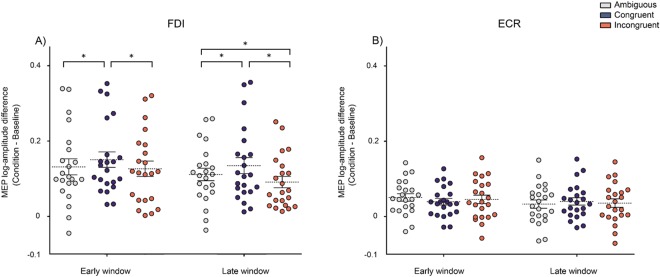
MEPs results. Amplitudes of MEPs recorded from the FDI (**a**) and ECR (**b**) muscles during the 3 action observation conditions (Congruent, Incongruent, and Ambiguous) at the 2 time-windows (early, late). Data points represent individual participants. Asterisks indicate significant comparison (*p* < 0.05). Error bars represent SEM and dotted lines MEAN.

In the later time-window (500 ms) after action onset, the facilitation effect for the FDI was still present (Congruent > Ambiguous, *p* = 0.0008), together with an inhibition of motor response, with lower normalized MEP amplitudes for actions observed within an incongruent context (M = 0.087, SD = 0.07) as compared to those observed within the congruent (M = 0.129, SD = 0.1; *p* = 0.0001) and ambiguous (M = 0.111, SD = 0.07; *p* = 0.0001) ones. No modulations were again observed for the ECR (all *p*s > 0.40). Please see Fig. 3.

### Correlation Results

**Figure 4:**
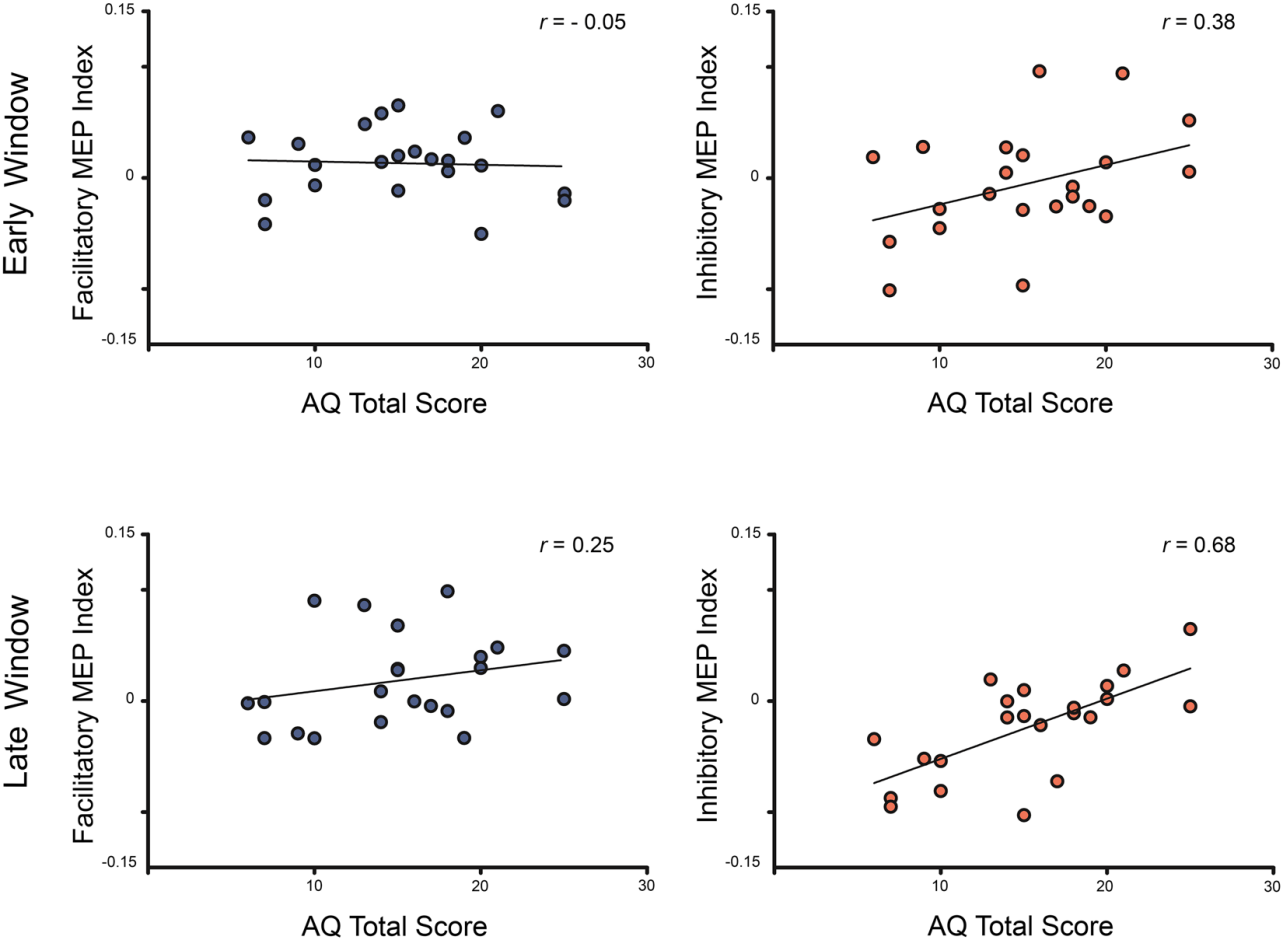
Pearson correlation results. Scatter plots illustrating the relationship between Facilitatory (Congruent – Ambiguous) and Inhibitory (Incongruent – Ambiguous) MEP indexes and the AQ total scores at the 2 time-windows (early, late).

We ran a Pearson’s correlation analysis to examine whether individual differences in ASD traits were associated with the facilitatory and/or inhibitory indexes. Please see Table 2 where the mean, standard deviation, and range for AQ scores are shown. No correlations were observed for the facilitatory index (Early time-window, *r* = −0.05, *p* = 0.82; Late time-window, *r* = 0.25; *p* = 0.26). However, the inhibitory index in the later time-window (500 ms) positively correlated with the AQ total score (*r* = 0.679; *p* = 0.001), showing that the higher the level of autistic traits the higher the level of motor responses for actions observed in incongruent contexts as compared to ambiguous ones. A post hoc power analysis conducted with the G*Power software^29^ using as input the results of the correlation between the inhibitory MEP index and the AQ in the late time window, showed that we reached a power of 0.95 with 22 participants. These results indicate that individuals exhibiting higher levels of autistic traits showed an impairment in inhibiting their motor responses to the observed kinematics when the context pointed to an incongruent overarching goal. In addition, no correlations were observed in the early time-window (*r* = 0.389; *p* = 0.07). See Fig. 4.

**Table 2:** Mean (SD) scores and range for the total AQ and AQ subscales.

	Mean	SD	Range
AQ Total	15.18	5.45	6–25
Social skills	2.09	1.50	0–7
Attention switching	4	2.02	1–8
Attention to detail	4.77	2.22	2–10
Communication	1.77	1.71	0–7
Imagination	2.27	1.48	0–6

Finally, no correlations were observed between the AQ total score and the *d* prime indexes (all *p*s > 0.11).

### Multiple Linear Regression Model

To identify the relative importance of factors contributing to the observed association between the autistic traits and the contextual modulation of motor responses, we used a multiple linear regression model with the 5 AQ subscales scores and gender as predictors and the inhibitory index as the dependent variable. The model explained a significant proportion of motor inhibition variability (adjusted R^2^ = 61%; *F* = 6.48; *p* = 0.002). The factors *Social skill* (β = 0.63, *p* = 0.002), *Gender* (β = 0.51, *p* = 0.008) and *Attention to detail* (β = 0.40, *p* = 0.01) showed significant regression coefficients. All other factors remained non-significant (all *p*s > 0.11). See Table 3 for further details. Overall, these results suggest that individuals with higher social skill deficits and greater detail-focused processing style are more impaired in inhibiting their motor responses when actions are observed in incongruent contexts. Furthermore, this impairment is greater in men as compared to women.

**Table 3:** Multiple regression analysis results.

Coefficients	B	Std. Error	Beta	t	*p*	Collinearity Statistics	
						Tolerance	VIF
Intercept	−0.137	0.022		−6.207	<0.001^*^		
Gender	0.045	0.015	0.519	3.046	**0.008** ^*^	0.640	1.564
Social skills	0.018	0.005	0.633	3.764	**0.002** ^*^	0.656	1.524
Attention switching	−0.006	0.004	−0.298	−1.681	0.113	0.589	1.697
Attention to detail	0.008	0.003	0.401	2.801	**0.013** ^*^	0.907	1.103
Communication	0.003	0.004	0.118	0.731	0.476	0.711	1.406
Imagination	−0.002	0.005	−0.083	−0.517	0.612	0.717	1.395

## Discussion

In the present study, we aimed to investigate the potential relationship between the autistic traits in a neurotypical sample and the motor resonance responses evoked by the observation of others’ actions embedded in naturalistic contexts.

At the neurophysiological level, we replicated previous findings^25,26^ by showing that the observation of movement kinematics embedded in congruent contexts led to an early facilitation of CSE, while the observation of the same movements in incongruent contexts resulted in a later inhibition of motor responses. Similar modulations were obtained at the behavioral level, with increased or decreased performance in kinematics recognition when actions were observed in congruent or incongruent contexts, respectively, as compared to ambiguous ones.

When correlating the AQ total score with the facilitatory and inhibitory MEP indexes, we found a specific and strong positive association between the level of autistic traits and the ability to inhibit M1 simulative responses if these where incompatible with the overarching intention cued by the context. Specifically, we found that individuals higher in autistic traits showed increased motor facilitation for actions embedded in incongruent as compared to ambiguous contexts, pointing to a failure in down-regulating M1 activity when a mismatch occurred.

Many studies have put the social skills impairments in ASD in relation to altered representation of other’s kinematics^10,11^. However, the origin of these deficits remains poorly understood, with studies either reporting preserved^13,14,30^ or abnormal MNS activity in ASD^10,11,15,16,31^. Here, in line with those studies showing no alteration of motor resonance in ASD, we found no correlation between autistic traits and motor facilitation during observation and prediction of actions embedded in ambiguous contexts (control condition). Furthermore, no correlations were observed between the AQ scores and performance in kinematics recognition, thus suggesting, in line with Bayesian accounts on ASD, that autistic-like observers are not impaired in coding perceptual kinematics *per se* but rather in combining it with context-based expectations^21^.

Importantly, however, autistic traits predicted an altered response to observed actions when these were embedded in incongruent contexts. This points to a specific alteration of the integration between perceptual kinematics and expectations of others’ intentions derived from the context.

Interestingly, our data show that autistic traits predicted the extent of inhibition of motor responses to observation of kinematics embedded in incongruent contexts, but not the facilitation in response to congruent ones. Thus, our data add to previous views on ASD by showing that autistic traits are specifically associated to the sensitivity of the motor system in mapping kinematics when they are incongruent with the observers’ expectations, rather than to a general deficit in coding these two information cues. In fact, facilitation for congruent contexts and inhibition for incongruent ones may reflect different mechanisms. In our previous studies, we found different time-courses in the motor responses associated to these effects, suggesting that while the early facilitation might reflect representation of the contextual expectations within the MNS, the later inhibition likely reflects modulation from higher cortical areas, beyond the MNS, which are call into play to solve the conflict^25,26^. Accordingly, during this later time-window, the ability to “mute” or silence the incompatible action representation was reduced in those individuals with higher autistic traits. Thus, our evidence points to alterations of later processes in the autistic-like brain, which fails to down-regulate M1 activity when the predicted action kinematics mismatches contextual expectations about others’ behaviors.

A core hypothesis derived from current Bayesian approaches to ASD is that sensory signals are weighted more highly when integrated with prior or contextual information (for a recent Review, see^32^). Our findings are in line with this suggestion but further specify that the greater reliance on sensory information mainly occurs in individuals with high levels of autistic traits when there is a mismatch between contextual information and sensory data. Indeed, if autistic traits were generally associated to greater weighting of sensory input we should have also observed a negative correlation between AQ-scores and the facilitatory MEP index, since individuals with higher levels of autistic traits should be less facilitated by congruent contexts, mostly relying on perceptual kinematics. Thus, according to our findings, the altered use of context-based expectations in ASD might be dependent on the degree of informativeness of the environment and/or on a failure in updating expectations when a discrepancy between what was expected and what was actually perceived (i.e., prediction errors) takes place.

Another important finding from our study came from the regression analysis, which showed that the model including the AQ-subscales and gender explained a significant proportion of motor inhibition variability, with social skill, attention to detail and gender being the regressors giving the greatest contributions. Thus, we found that the differential inhibition of M1 was related to individual differences in ASD-like traits associated to both social (i.e., social skill) and non-social (i.e., attention to detail) aspects. Furthermore, these associations were independent from the observer’s gender, which showed an independent effect with greater impairments in men than women. This later finding is in line with previous studies using the AQ and showing consistent gender differences, such that neurotypical men obtain significantly higher AQ scores than neurotypical women^33,34^.

While the correlations with social impairments and gender were expected, the finding of an independent influence on motor inhibition from the attention to detail subscale deserves further discussion. This subscale measures non-social aspects related to perceptual atypicalities. Indeed, a perceptual phenomenon that has been repeatedly associated to ASD is the presence of a local processing bias, which seems to be independent of social functioning^35^ and theory of mind abilities^36^. In other words, individuals with ASD perceive the world differently, exhibiting a detail-focused style when processing visual information. Interestingly, evidence from a recent meta-analysis^37^ suggests that the altered perceptual organization in ASD may be related to a shift towards slower global processing. In this view, perceptual atypicalities would be mainly related to timing alterations, with ASD individuals being slower in global processing as compared to neurotypical controls. Furthermore, it has been shown that differences in the temporal interplay between local and global processing particularly affect the construction of dynamic visual representations^38^. For instance, when the time to integrate a motion signal is short, ASD perceptual deficits become more pronounced, suggesting that global motion processing in ASD evolves more slowly over time^39^. Within this context, our finding of an independent contribution of the attention to detail subscale could be related to a delayed integration of the dynamic relationships between the contextual and the kinematics cues. This temporal delay might explain the absence of down-regulation of M1 activity in the late time window and opens the possibility that inhibition for incongruent contexts could actually occur but in a later time-window. However a limitation of our experimental design is that CSE was measured up to 500 ms after action onset, not allowing us to test this hypothesis.

Another major limitation is the small sample size for a correlational design (*n* = 22), which requires caution when generalizing and interpreting current results. However, the post hoc analysis showed a more than acceptable power for our study^40^. It is worth noting that previous studies^41,42^ measuring MEPs in neurotypical individuals and correlating motor activity with AQ scores have used smaller samples. Nevertheless, further research is clearly required to extend current findings to larger population.

## Conclusions

Collectively, our findings suggest that autistic traits might be related to differences in the extent to which individuals are able to integrate context-based expectations and current sensory evidence. More specifically, our finding of an association between autistic traits with inhibition for incongruent contexts -but not with facilitation for congruent ones-, points to a specific difficulty in integrating perceptual information and expectations derived from the context when a mismatch occurs. Overall, these findings are in line with current proposals on ASD speaking in favor of an abnormal interplay between simulative motor mapping of low-level movement kinematics in the MNS and top-down signals from areas beyond this network during action prediction and recognition.

## Methods

### Participants

Twenty-two individuals recruited at the University of Udine took part in the study (13 women, M = 22.9, SD = 3.06). Participants were all right-handed according to the Standard Handedness Inventory^43^, had normal acuity in both eyes and were free from any contraindication to TMS^44^. They gave their written informed consent before the beginning of the experiment and received course credits for participating in the study. The experimental procedures were approved by the local Ethics Committee (Comitato Etico Regionale Unico, Friuli Venezia Giulia, Italy) and were carried out in accordance with the ethical standards of the Declaration of Helsinki. None of the participants reported history of neurological, psychiatric, or other major medical problems. No discomfort or adverse effects during TMS were reported or noticed in any of the participants.

After the experiment, participants completed the Italian version^45^ of the Autism Spectrum Quotient (AQ)^28^. The AQ is a self-report questionnaire which quantifies the degree to which individuals with normal intelligence have the traits associated to the autistic spectrum (social abnormalities, communication difficulties, and stereotyped and rigid behaviour), via assessing five different factors: *Social skills*, *Attention switching*, *Attention to detail*, *Communication* and *Imagination*. Each factor is assesed by 10 items and higher scores are obtained by individuals endorsing more autistic-like behaviour.

### Stimuli and Task

Videos were taken from Amoruso and Urgesi (2016). They were recorded in color with a Canon EOS 550D camera at 30 frames per second and were further edited with Adobe Premiere Pro CS3 3.0. Length was matched across videos so that they had an equivalent duration (15 frames for a total of 500 ms). All videos depicted a female model performing reach-to-grasp movements of different objects with her right hand. Depending on the kinematics (precision vs whole-hand grips), each object could be grasped to perform either one of two possible actions. For instance, in the case of the object “mug”, the actions were a) to drink and b) to clean, each of them performed with their correspondent kinematics: reaching-to-grasp the mug by its handle using a precision grip, and reaching-to-grasp the mug from the top using a whole-hand grip, respectively^46^.

Each action was recorded in 3 different contexts: congruent, incongruent, and ambiguous. In the congruent condition, the action suggested by the context was compatible with the action suggested by the kinematics. Conversely, in the incongruent condition, the context interfered with the kinematics by cueing to the opposite action. Finally, in the ambiguous condition, contextual cues were not informative about which action was likely to unfold (see Fig. 1 and Videos 1–3 for examples of each condition). For a complete description of objects, action labels, grips, contexts, and their possible combinations, please refer to Amoruso and Urgesi^27^.

Stimuli were validated in a previous study^27^, which confirmed the appropriate manipulation of the plausibility of each action when embedded in congruent, ambiguous, and incongruent contexts (with parametrically decreasing levels of plausibility, respectively). Moreover, to ensure that the observed motor modulations were actually due to the manipulation of the contextual information and not to differences in the movement kinematic profiles of the same action across scenes, we performed a frame-by-frame kinematic analysis of the videos. This analysis showed that kinematics were comparable across contexts^25^.

In a two-alternative forced choice (2AFC) task, participants were requested to watch the videos and predict action unfolding. A temporal occlusion paradigm was used, with the videos being stopped before the model made contact with the object. Note that, by doing this, participants were able to observe the pre-shaping of the hand configuration during the reaching-to-grasp phase of the movement but not the grasping movement itself. To build-up their predictions, participants were instructed to pay attention to both aspects of the scene: the kinematic information conveyed by the model’s hand and the contextual information in terms of objects configurations. The inclusion of ambiguous trials, in which the context was not informative, prevented participants from focusing attention only on the contextual cues when giving their responses.

### Electromyography (EMG) Recording and spTMS

Single-pulse TMS was applied to the left M1 using a Magstim 200 stimulator (maximum output = 2T at coil surface, pulse duration = 250 μsec, rise time = 60 μsec, The Magstim Company, Carmarthenshire, Wales, UK) connected to a 70-mm figure-of-eight coil (Magstim polyurethane-coated coil). MEPs were recorded simultaneously from the First Dorsal Interosseous (FDI) and from the Extensor Carpi Radialis (ECR, control muscle) of the right hand during task performance. Muscle selection was based on previous findings using the current paradigm^25–27^ and evidence from spTMS studies showing that while both muscles are synergistically activated during the observation of reach-to-grasp movements^47^, only the FDI corticospinal representation is differently modulated by the observation of precision vs. whole-hand grips^48,49^. Thus, we expected a greater involvement of the FDI muscle as compared to the ECR. Surface Ag/AgCl disposable electrodes (1 cm diameter) were placed in a belly-tendon montage for each muscle. The EMG signal was amplified, filtered (band-pass 5 Hz to 20 kHz) and recorded with the Biopac MP-36 system (BIOPAC Systems, Inc., Goleta, CA) at a sampling rate of 50 kHz.

The coil was positioned tangentially on the scalp, with the handle pointing backward and approximately 45° lateral from the midline, perpendicular to the line of the central sulcus^50^. This orientation was chosen based on the finding that the lowest motor threshold is achieved when the induced electric current in the brain is flowing perpendicular to the central sulcus^51,52^. The optimal scalp position (OSP) for inducing MEPs in both muscles was detected by moving the coil in 1-cm steps over the left M1 and by delivering TMS pulses at constant intensity until the largest MEPs for the FDI were found. Then, the OSP was marked with a pen on a tight-fitting bathing cap worn by participants. The coil was held on the scalp by a holder with an articulated arm, and its position with respect to the mark was continuously checked to compensate for small movements of the participants’ head.

The intensity of magnetic pulses was set at 120% of the individual resting motor threshold (rMT), defined as the minimum intensity of the stimulator output able to produce MEPs with amplitudes of at least 50 μV with 50% probability in higher threshold muscle (Rossini *et al*. 1994) in 5 out of 10 consecutive pulses. The rMT ranged from 38% to 59% (M = 46%, SD = 5.4%). To ensure that there was no unwanted background EMG activity before the magnetic pulse, the signal from both muscles was continuously verified online by visually monitoring the EMG signal. MEPs’ peak-to-peak amplitudes (in mV) were collected and stored in a computer for off-line analysis.

### Trial Structure

Stimuli were presented using E-prime V2 software (Psychology Software Tools) on a 21 inch CRT monitor (resolution, 1024 × 768 pixels; refresh frequency, 60 Hz). Participants sat in a comfortable armchair in a dimly lit room ~1 m away from the monitor with prone hands resting on a pillow. They were instructed to pay attention to the displayed stimuli and to avoid moving their right hand. Videos appeared at the center of the screen on a neutral background (subtending ~ 15.96° × 11.97° of visual angle). Trials started with a visual warning cue lasting for 5 s (the Italian word “*attendi*”, in English “attention”), followed by the video presentation lasting 500 ms. TMS pulses were delivered online at either 300 ms or 500 ms after action onset. After the video, a frame was presented with the verbal descriptors of the two possible goals (e.g., “*versare*” and “*spostare*”, in English “to pour” and “to place” respectively; one located up and the other located down) written in black on a white background. This frame remained on the screen until a response was recorded. Participants verbalize their responses (by saying “*su*” or “*giù*”, in English “up” or “down”, respectively) and the experimenter recorded the answer. The location of the descriptors was counterbalanced, ensuring that in half of the trials one of the descriptors was presented up and, in the other half, it was presented down. This procedure enabled us to prevent participants from planning their response on the basis of the descriptors’ spatial location. Verbal responses were used to prevent that peripheral muscular contraction artifacts resulting from button press contaminated MEPs. Importantly, verbal responses were required after the TMS pulse was delivered^53^. A total of 42 videos (14 actions embedded in three different contexts) were used. Each video was presented twice, resulting in 84 stimuli randomly presented in two blocks of 42 trials each. Prior to video presentation, baseline corticospinal excitability was assessed by acquiring 10 MEPs while participants passively watched a fixation cross.

### Data Analysis

#### Behavioral data

Individual performance expressed as *d* prime values (*d*’), a bias-corrected measure of sensitivity in discriminating between 2 categories^54^, was calculated for each condition. In the *d*’ analysis, ‘precision grips’ identified as ‘precision grips’ were considered as “hits” and ‘whole-hand grips’ identified as ‘precision grips’ were considered as “false alarms”. More specifically, correct responses were defined by the kinematics, not by the context. The *d*’ values were calculated by transforming the response proportion to *z*-scores, and then subtracting the *z*-score that corresponds to the false-alarm rate from the *z*-score that corresponds to the hit rate^55^. The *d*’ values were subjected to a repeated-measures analysis of variance (RM-ANOVA) with Time (early, late) and Context (congruent, incongruent, and ambiguous) as within-subject variables.

#### MEP data

Individual mean peak-to-peak (in mV) amplitudes of MEPs recorded from the FDI and ECR muscles were calculated separately for each condition. Since background EMG is known to modulate MEP amplitude, it was assessed in each participant by calculating the mean rectified EMG signal across a 100 ms interval prior to TMS. MEPs with preceding background EMG deviating from the mean by >2 SD were removed from further analysis. The total percentage of excluded MEPs (FDI, 300 ms: congruent, 5.84%; incongruent, 6.16%; ambiguous, 7.79%; FDI, 500 ms: congruent, 8.44%; incongruent, 8.76%; ambiguous, 6.16%; ECR, 300 ms: congruent, 6.49%; incongruent, 7.46%; ambiguous, 9.09%; ECR, 500 ms: congruent, 6.81%; incongruent, 8.76%; ambiguous, 8.11%) did not differ between conditions and muscles (all *F*s < 1.33; all *P*s > 0.27). Furthermore, after removing contaminated trials, at least 10 MEP trials (>70%) remained in each experimental condition for all participants.

Raw MEP values were log-transformed to address non-normality resulting from positive skew^56^. MEP amplitudes recorded during action observation conditions were expressed as difference values from MEPs recorded during baseline. These normalized action-observation MEPs were analyzed by means of a 3-way RM-ANOVA with Muscle (FDI, ECR), Time (early, late) and Context (congruent, incongruent and ambiguous) as within-subjects’ variables. Estimates of the effect size were obtained using the partial eta-squared. Post-hoc analysis was carried out using the Newman-Keuls test. The analyses were implemented in Statistica software v.10 (Statsoft, Tulsa, OK) and α value was set at 0.05.

#### Correlation Analysis

First, we applied the Shapiro-Wilk test to determine the distribution of the variables included in the correlational analysis. Importantly, all the variables showed a normal distribution (all *p*s > 0.10). Thus, we calculated Pearson correlation coefficients between the AQ-scores and the mean MEP values obtained in the ambiguous condition (control condition). Since no correlations were found (Early time-window, *r* = −0.145, *p* = 0.51; Late time-window, *r* = −0.208; *p* = 0.35), we computed two MEP indexes obtained by subtracting, for each subject, the mean normalized MEPs value for the congruent minus the ambiguous condition (facilitatory MEP index) and the incongruent minus the ambiguous condition (inhibitory MEP index). Briefly, these indexes reflect that the higher the facilitatory MEP index, the higher the facilitation for congruent contexts and the higher the inhibitory index, the lower the inhibition for incongruent contexts, respectively. We then calculated Pearson correlations between the individual AQ scores and the facilitatory and inhibitory MEP indexes in the early and late time windows. A Bonferroni correction prcedure was applied to control for multiple comparisons (α = 0.012; 4 comparisons).

An analogue procedure was performed for the *d* prime values. Since no correlations were found for the ambiguous conditions and the AQ scores at the behavioral level (Early time-window, *r* = −0.167, *p* = 0.45; Late time-window, *r* = −0.326; *p* = 0.13), the two indexes for the early and late time windows were computed and correlated separately.

#### Multiple Linear Regression Analysis

Finally, we conducted a multiple linear regression analysis to identify factors that may contribute to the MEP effects. We followed standard procedures for assessing multicollinearity and confirmed that tolerance (all values < 0.9) and variance inflation factors (all VIF < 1.69) were low, overall indicating that multicollinearity did not affect our statistical analysis.

The five AQ subscales were entered as predictors together with gender, since it has been shown that gender differences in autistic traits are present in the nonclinical population, with men scoring significantly higher than women^24^.

## Electronic supplementary material

To drink_Ambiguous

To drink_Congruent

To drink_Incongruent

## Data Availability

The datasets used in the current study are available from the corresponding author on reasonable request.
